# Family functioning, attachment security, and psychological stress among university students attending a psychological counselling service: an exploratory cluster analysis

**DOI:** 10.1186/s40359-026-05192-z

**Published:** 2026-07-20

**Authors:** Marco Cacioppo, Andrea Fontana, Eleonora Topino, Simona De Stasio, Giuseppe Mannino, Maria Concetta Pellicciari, Lucia Sideli

**Affiliations:** 1 Department of Human Sciences, LUMSA University of Rome, Via della Traspontina 21, Rome, 00193 Italy; 2 Department of Theoretical and Applied Sciences, eCampus University, Novedrate, Italy; 3 Department of Law, Economics, and Communication, LUMSA University, Via Filippo Parlatore, 65, Palermo, 90141 Italy

**Keywords:** University students, Distress, Family functioning, Attachment, Cluster analysis

## Abstract

**Background:**

Psychological stress is highly prevalent during higher education studies but mental health trajectories are shaped by risk and protective factors. Attachment security and balanced family functioning might reduce students’ psychological stress but literature is still limited. The aim of this study was to investigate the relationship between psychological stress, family functioning, and adult attachment in order to identify more vulnerable groups.

**Method:**

A sample of university students self-referring to an on-campus counselling service (*n* = 171; 81.9% female and 18.1% male; *Mean age* = 22.53 years, *SD* = 3.35) was assessed for psychological stress, family functioning, and adult attachment. A Two-Step Cluster Analysis and ANOVA were conducted to identify cluster groups reflecting distinct profiles and to test between-group differences.

**Results:**

Sixty-three percent (63.2%) scored above the CORE-OM clinical cut-off. Distress correlated positively with unbalanced family patterns (Disengaged, Enmeshed, Rigid, Chaotic) and with Attachment Anxiety and Avoidance; and negatively with Cohesion, Flexibility, Satisfaction, and Communication. A two-cluster solution emerged: (1) High Distress / Low Relational Functioning with lower balanced functioning, higher unbalanced patterns, and higher attachment insecurity; and (2) Low Distress / High Relational Functioning with the complementary pattern.

**Conclusions:**

Student distress was systematically intertwined with family functioning and attachment, and coalesced into two clinically meaningful profiles. Findings support mechanism-focused, stepped care in campus services: low-distress students may benefit from low-intensity psychoeducation and skills rehearsal, whereas high-distress students may require brief, structured modules targeting communication, boundary-setting, and emotion regulation, with referral pathways activated when complexity or acute risk is present.

## Introduction

University students’ mental health has received greater attention over the past years [1–3]. Recent Italian evidence has also emphasized the importance of assessing well-being, ill-being, and health-related lifestyle behaviours among university students, further supporting the need for systematic monitoring in higher education settings [4]. According to a recent systematic review, an average of 13% of students have experienced severe depression, 17% severe anxiety, 18% disordered eating, 25% post-traumatic stress symptoms, 40% sleep disorders, and 3 to 20% suicide-related thoughts or behaviours [5]. Beyond full-blown mental health disorders, a relevant proportion of university students have referred psychological stress during their degree courses, as expressed by emotional, behavioural, or relational problems, and self- or other-directed aggressive behaviours [6, 7].

In the past ten years, systematic reviews and large epidemiological surveys in the UK and the US have documented an increase of depression, anxiety, and suicidality, particularly among women [5, 8, 9]. Following the COVID-19 pandemic, a greater proportion of both medical and non-medical students have shown mental health problems related to social isolation and quarantine, academic stress, financial difficulties, exposure to COVID-19, reduced physical exercise, and excessive Internet use [10, 11]. More frequent mental health problems were depression, anxiety, post-traumatic, and sleep symptoms [12–14] but also included behavioural addictions and disordered eating [15].

The rise of mental health problems among university students is also documented by the fact that, according to a recent meta-analysis, 35% have sought any type of support both inside and outside university, with 21% of them accessing outpatient services and 1–3% to inpatient services while attending their undergraduate or postgraduate degree [16]. In the Italian university context, recent studies on psychological support and counselling services have similarly emphasized the importance of monitoring students’ mental-health trajectories and evaluating the effectiveness of university-based interventions [17, 18]. Furthermore, this data may be underestimated, due to the consistent barriers preventing students’ help-seeking behaviours, such as social stigma, poor mental health literature, and excessive self-reliance [19].

### Risk and protective factors of students’ mental health

Trajectories of mental health problems of university students are shaped by individual- and social-level factors. At individual level, psychiatric comorbidities, disabilities, maladaptive coping strategies (e.g., rumination), poor mental health literacy, and high academic load have been associated with more severe psychological stress and negative mental health outcomes [6, 7, 20, 21]. At social level, minority status (e.g., ethnic minority or LGBTQ), parental separation, parental mental health problems, childhood maltreatment, recent life events and current problems (e.g., sexual harassment, financial difficulties) have been related to negative mental health trajectories [20, 21].

Several factors have shown a protective role for well-being and mental health. As pointed out by a recent systematic review, higher engagement with academic and leisure activities, self-esteem, and personality traits fostering adjustment to life changes and challenges – such as emotion regulation, persistence, flexibility, and resilience - are associated with greater well-being and satisfaction [20]. At social level, being involved in supportive and reliable social networks and perceived parental acceptance were found to be protective factors against psychological distress [20]. Evidence suggests that social support might act both directly, by reducing psychological stress, and indirectly, by fostering self-esteem and resilience, which in turn improve students’ well-being [22]. Consistently, university-based initiatives focused on self-care and stress management have been proposed as promising strategies to support students’ well-being, particularly among doctoral students [23].

### Theoretical background

From a developmental-relational perspective, psychological stress during the transition to university can be conceptualized as the outcome of dynamic transactions between individual regulatory capacities and relational contexts, rather than as a purely intrapsychic condition [24–26]. This view is consistent with developmental psychopathology models, which emphasize that adaptation and maladaptation emerge over time through the interplay of individual, interpersonal, and contextual processes [27, 28]. University students are typically in emerging adulthood, a developmental period characterized by identity exploration, increasing autonomy, and the renegotiation of dependence on parents and significant others [29]. In this phase, family functioning may continue to operate as a central relational context for emotional regulation, support-seeking, and the management of separation and autonomy. Empirical evidence suggests that students’ perceptions of their family environment are linked to adjustment during the college transition, and that emotion coping may partly explain how family functioning relates to academic, social, and personal-emotional adaptation [30]. Similarly, family communication patterns may influence first-year college adjustment through perceived family support and the quality of parental advice [31]. Attachment security provides a complementary explanatory mechanism: secure parental attachment has been associated with healthier separation-individuation and better college adjustment, supporting the idea that autonomy develops most adaptively within reliable relational bonds rather than through relational disengagement [32]. Accordingly, psychological stress, family functioning, and attachment security can be regarded as interconnected dimensions of the same developmental-relational system, in which students’ current distress reflects not only exposure to academic or social demands, but also the quality of relational resources and internalized expectations through which support, autonomy, and affect regulation are negotiated.

### The role of attachment security and family functioning

Given the developmental challenges posed by adjusting to the higher education system, academic load, and often separation from parents and friends, attachment security and positive family functioning might play a significant role in students’ psychological well-being.

Attachment security is defined as the capacity to rely on protective figures when experiencing physical or psychological distress, which is rooted in childhood consistent experiences of responsive and attentive care from parents [33, 34]. Individuals with attachment security present a positive image of self and others and use adaptive emotion regulation strategies, based on search for support from caregivers and from significant others [35, 36]. By contrast, attachment insecurity is related to a negative image of the self or/and the others, minimization or amplification of psychological distress, and maladaptive emotion regulation strategies, which increase the risk for psychopathology [37]. Accumulating evidence has shown that attachment insecurity predicted greater psychological distress both at the beginning and during students’ academic careers, whereas attachment security was related with better mental health by promoting search for social support and resilience [38–41].

Family functioning is defined as the overall quality of family life, which may be characterized in terms of emotional closeness, adaptability to life changes, good communication, and relational satisfaction [42]. Family functioning and attachment security are bi-directionally related [43], as attachment security fosters authentic communication, trust in others, acceptance and respect among family members and, conversely, adaptive family functioning is related to secure bonds [44]. Literature suggests that perceived parental acceptance and support are associated with students’ adjustment to higher education challenges and well-being [45, 46], whereas parental over-protection and over-indulgence predicted maladaptive coping strategies and mental health problems [47]. Moreover, family cohesion and flexibility have been associated with lower prevalence of pathological addictive behaviours (such as gambling, problematic smartphone use, or compulsive shopping) among young adults [48–50]. However, to the best of our knowledge, the relationship between psychological stress, family functioning, and attachment security among university students has not been investigated.

### The present research

The general aim of this study was to explore the psychological functioning of university students accessing the university psychological counselling service, with particular attention to the role of family functioning and adult attachment patterns. Drawing on existing literature that emphasizes the interplay between psychological distress, family dynamics, and attachment styles (e.g [41, 46]), , the aim of the research was to explore the presence of distinct psychological profiles among students based on their levels of psychological distress, specifically focusing on the role of family functioning and attachment style.

## Method

### Design, participants, and procedure

The study involved the assessment of a specific population at a single time point, without manipulating any variables or implementing an intervention. Therefore, a quantitative, observational, cross-sectional design was adopted. A non-probabilistic convenience sampling method was used. Participants were 171 university students who accessed the University’s Psychological Counselling Service of LUMSA University. They ranged in age from 18 to 39 years (*Mean age* = 22.53 years, *SD* = 3.35), and the majority were female (81.9%, *n* = 140), while 18.1% were male (*n* = 31). The inclusion criteria were as follows: being enrolled as a university student at LUMSA University, having accessed the University’s Psychological Counselling Service, having completed an initial consultation with a licensed psychologist from the counselling center, and having provided informed consent to participate in the study. No exclusion criteria were applied on the basis of gender, degree program, psychological symptoms, or previous psychological or psychiatric treatment. To access the counselling service, students were required to submit a request via the service’s official email address using their institutional university account. The University’s Psychological Counselling Service served as the recruitment setting for the study. Data collection was conducted exclusively through an online questionnaire administered via Google Forms after the initial consultation with a licensed psychologist from the counselling center. Students received the link to the questionnaire via email and completed it through Google Forms. The questionnaire was accompanied by an electronic informed consent form, which had to be accepted before proceeding. In order to submit the survey, participants were required to complete all sections of the form, which ensured that no missing data were present. Participation was voluntary; students were free to interrupt the process at any time, and completing the survey did not imply any obligation to continue with counselling sessions.

### Measures

####  The Clinical Outcomes in Routine Evaluation Outcome Measure (CORE-OM)

The CORE-OM (Evans et al. [51]; Italian version: Palmieri [52]; Palmieri et al. [53]) is a 34-item self-report measure of psychological functioning. Items are on a 5-point Likert scale, from 0 (*Not at all*) to 4 (*Most or all of the time*). Both a total score of distress and 4 subscales may be calculated: (1) well-being, concerning overall well-being (this is the only domain interpreted in reverse: higher scores indicate lower well-being, and it reflects overall well-being); (2) problems, which focuses on emotional, behavioural, or relational difficulties; (3) functioning, which refers to daily functioning, including the ability to carry out daily activities, maintain social or work relationships, and engage in social activities; (4) risk, which focuses on risky behaviours, such as self- or other-directed harm. For the total score, gender-specific cut-offs are available to distinguish between low levels of psychological distress and clinically significant distress [52, 53]. In the present sample, the CORE-OM showed satisfactory internal consistency (total score, α = 0.92; well-being, α = 0.70; problems, α = 0.91; functioning, α = 0.72; risk, α = 0.61).

#### The Family Adaptability and Cohesion Evaluation Scales – IV (FACES-IV)

The FACES-IV (Olson [54]; Italian version: Baiocco et al. [55]) is a 62-item self-report instrument, designed to assess family functioning according to the Circumplex Model of Marital and Family Systems [42, 54]. Items are rated on a 5-point Likert scale, from 1 (*strongly disagree*) to 5 (*strongly agree*). The FACES-IV includes six subscales assessing both balanced and unbalanced family dynamics: (1) cohesion, reflecting emotional closeness and involvement among family members; (2) flexibility, assessing the family’s capacity to adapt to changes; (3) disengaged, indicating emotional distance and low connectedness; (4) enmeshed, representing excessive closeness and blurred boundaries; (5) chaotic, related to unpredictability and lack of structure; and (6) rigid, referring to inflexibility and resistance to change. In addition, the instrument includes two supplementary 10-item scales: the Family Communication Scale, measuring the quality of interpersonal communication within the family, and the Family Satisfaction Scale, assessing overall satisfaction with family functioning. In the present study, the Italian version of the FACES-IV showed satisfactory internal consistency (cohesion, *α* = 0.85; flexibility, *α* = 0.75; enmeshed, *α* = 0.69; disengaged, *α* = 0.74; chaotic, *α* = 0.66; rigid, *α* = 0.66; communication, *α* = 0.93; satisfaction, *α* = 0.95).

#### The Relationship Questionnaire (RQ)

The Relationships Questionnaire (RQ; Bartholomew & Horowitz [56]; Italian version: Carli [57]) is a 4-item self-report instrument designed to assess adult attachment styles. Each item corresponds to one of four prototypical patterns: secure, preoccupied, dismissing, and fearful attachment. Respondents rate the extent to which each description matches their typical relational style using a 7-point Likert scale, from 1 (*It does not describe me at all*) to 7 (*It very much describes me*). Based on responses to these four items, it is possible to compute continuous scores reflecting two broader attachment dimensions: attachment anxiety and attachment avoidance. Due to the nature of the scoring procedure, which relies on the combination of single-item ratings through a predefined algorithm, internal consistency (e.g., Cronbach’s alpha) cannot be calculated.

### Data analysis

All the statistical analyses were performed using SPSS (v. 21.0; IBM, New York, USA). Descriptive statistics and Pearson’s *r* correlations were calculated. To identify potential subgroups within the sample, a Two-Step Cluster Analysis was conducted [58, 59]. This method was selected because it allows the identification of naturally occurring profiles based on multiple input variables, including both categorical and continuous indicators [60]. In the present study, the number of clusters was fixed a priori to two, in line with the study aim of distinguishing students with different psychological and relational profiles, i.e., students with low psychological distress and more adaptive relational functioning versus students with clinically significant psychological distress and less adaptive relational functioning. This two-cluster solution was chosen to obtain a parsimonious and clinically interpretable distinction between students showing a more adaptive profile and those showing a more vulnerable profile, consistent with the use of the CORE-OM clinical cut-off as one of the input variables. The cluster solution was obtained using log-likelihood as the similarity measure. The input variables included the dichotomous CORE-OM clinical cut-off variable [41–43], the family functioning dimensions from the FACES-IV [54, 55] and attachment patterns as measured by the RQ [56, 57]. The emerging groups were not created manually or by using a single criterion, but were derived from the combined configuration of psychological distress, family functioning, and attachment-related variables. Among the variables entered into the cluster analysis, no correlation coefficient was ≥ 0.90, thus excluding serious multicollinearity issues [61]. The quality and interpretability of the cluster solution were evaluated by examining the ratio of cluster sizes, with a threshold below 3.0 considered acceptable [62]. The silhouette measure of cohesion and separation was also examined, with values above 0.2 considered adequate for exploratory purposes [63]. After cluster membership had been assigned, the two groups were compared using one-way Analyses of Variance (ANOVAs) on the continuous variables included in the cluster solution. These post-hoc comparisons were conducted to describe the specific profile of each cluster and to verify whether the two groups differed significantly across the psychological, family, and attachment-related dimensions considered in the study. This procedure is a common and recommended approach to evaluate the internal coherence and external validity of clustering results [58, 63].

## Results

Based on the CORE-OM cut-off scores, descriptive statistics indicated that most students (63.2%) reported clinically significant levels of psychological distress. Pearson’s *r* correlations showed significant associations between the variables of interest (see Table 1).

**Table 1:** Correlation Matrix

	1	2	3	4	5	6	7	8	9	10	11	12	13	14	15
Outcomes															
1. Total scores	1														
2. Well-Being	**0.838** ^**^	1													
3. Problems	**0.942** ^**^	**0.728** ^**^	1												
4. Functioning	**0.846** ^**^	**0.673** ^**^	**0.653** ^**^	1											
5. Risk	**0.516** ^**^	**0.373** ^**^	**0.466** ^**^	**0.297** ^**^	1										
*Family Functioning*															
6. Cohesion	− 0.422**	− 0.359**	− 0.350**	− 0.428**	− 0.223**	1									
7. Flexibility	− 0.390**	− 0.385**	− 0.291**	− 0.432**	− 0.157*	0.771**	1								
8. Disengaged	0.297**	0.283**	0.229**	0.309**	0.176*	− 0.642**	− 0.493**	1							
9. Enmeshed	0.218**	0.200**	0.201**	0.180*	0.117	0.063	− 0.019	− 0.039	1						
10. Rigid	0.159*	0.062	0.169*	0.150	0.041	− 0.112	0.015	0.174*	0.392**	1					
11. Chaotic	0.291**	0.321**	0.225**	0.291**	0.123	− 0.260**	− 0.285**	0.402**	0.177*	− 0.063	1				
12. Family Communication	− 0.452**	− 0.404**	− 0.367**	− 0.460**	− 0.243**	0.780**	0.750**	− 0.610**	0.009	− 0.176*	− 0.294**	1			
13. Family Satisfaction	− 0.405**	− 0.349**	− 0.317**	− 0.441**	− 0.205**	0.731**	0.750**	− 0.643**	− 0.054	− 0.238**	− 0.342**	0.837**	1		
*Attachment*															
14. Attachment Anxiety	**0.169** ^*^	0.107	**0.167** ^*^	**0.162** ^*^	0.034	− 0.109	− 0.102	0.073	0.071	− 0.072	− 0.017	− 0.132	− 0.108	1	
15. Attachment Avoidance	**0.235** ^**^	**0.195** ^*^	0.145	**0.347** ^**^	0.029	**− 0.261** ^**^	**− 0.179** ^*^	**0.165** ^*^	0.025	0.148	0.137	**− 0.238** ^**^	**− 0.246** ^**^	− 0.054	1

Values in boldface indicate statistically significant results

* Correlation is significant at the 0.05 level (2 tailed)

** Correlation is significant at the 0.01 level (2-tailed)

The CORE-OM total score was significantly and positively correlated with all CORE-OM subscales (*p* < .01), including well-being (*r* = .838), problems (*r* = .942), functioning (*r* = .846), and risk (*r* = .516). It was also positively associated with unbalanced family functioning factors, including disengagement (*r* = .297, *p* < .01), enmeshment (*r* = .218, *p* < .01), rigidity (*r* = .159, *p* < .05), and chaos (*r* = .291, *p* < .01). Moreover, the CORE-OM total score was positively correlated with insecure attachment dimensions, namely attachment anxiety (*r* = .169, *p* < .05) and attachment avoidance (*r* = .235, *p* < .01). Finally, the CORE-OM total score was significantly and negatively associated with family cohesion (*r* = –.422, *p* < .01), flexibility (*r* = –.390, *p* < .01), family satisfaction (*r* = –.405, *p* < .01), and family communication (*r* = –.452, *p* < .01).

The Two-Step Cluster Analysis yielded a two-cluster solution based on 11 input variables, including the CORE-OM dichotomous variable, family functioning, and attachment patterns. Cluster sizes were moderately balanced, with Cluster 1 including 108 participants (63.2%) and Cluster 2 including 63 participants (36.8%). The ratio of the largest to the smallest cluster was 1.71, below the recommended threshold of 3.0, suggesting adequate distribution and interpretability of the grouping. The first group was characterized by psychological distress levels above the clinical cut-off and a less adaptive relational profile. Students in this cluster scored higher on psychological distress (*M* = 1.78, *SD* = 0.496), lower on balanced family functioning dimensions, including cohesion (*M* = 22.19, *SD* = 6.455), flexibility (*M* = 20.50, *SD* = 5.635), satisfaction (*M* = 26.91, *SD* = 10.334), and communication (*M* = 28.69, *SD* = 10.188), and higher on unbalanced patterns, including disengagement (*M* = 18.34, *SD* = 5.652), enmeshment (*M* = 16.04, *SD* = 5.103), chaos (*M* = 17.42, *SD* = 5.535), and rigidity (*M* = 17.91, *SD* = 5.156). They also reported higher levels of insecure attachment, with higher scores on attachment anxiety (*M* = 0.40, *SD* = 2.864) and attachment avoidance (*M* = 2.08, *SD* = 3.908). Based on this profile, this cluster was labelled as “High Distress / Low Relational Functioning.” The second group was characterized by psychological distress levels below the clinical cut-off and a more functional interpersonal profile. Students in this cluster showed lower psychological distress (*M* = 0.80, *SD* = 0.225), higher levels of balanced family functioning, including cohesion (*M* = 26.94, *SD* = 5.111), flexibility (*M* = 24.41, *SD* = 4.868), satisfaction (*M* = 34.24, *SD* = 8.676), and communication (*M* = 36.19, *SD* = 8.189), and lower levels of unbalanced patterns, such as disengagement (*M* = 15.43, *SD* = 4.518), enmeshment (*M* = 13.65, *SD* = 4.159), chaos (*M* = 15.14, *SD* = 4.204), and rigidity (*M* = 16.27, *SD* = 4.209). They also reported lower attachment anxiety (*M* = -0.65, *SD* = 3.143) and attachment avoidance (*M* = -0.02, *SD* = 3.722). This cluster was labelled as “Low Distress / High Relational Functioning.”

Comparison analyses of the one-way ANOVA showed significant differences between the two groups (“Low Distress / High Relational Functioning” and “High Distress / Low Relational Functioning”) across all variables examined (see Table 2).

**Table 2:** Means, Standard Deviations, and ANOVA Comparisons between the clusters “High Distress / Low Relational Functioning” and “Low Distress / High Relational Functioning”

Variable	Cluster 1 (*N* = 108)		Cluster 2 (*N* = 63)		F (1,169)	*p*
	M	SD	M	SD		
CORE-OM Total Score	1.780	0.496	0.800	0.225	219.628	< 0.001
*Family Functioning*						
Cohesion	22.190	6.455	26.940	5.111	24.976	< 0.001
Flexibility	20.500	5.635	24.410	4.868	21.155	< 0.001
Disengaged	18.340	5.652	15.430	4.518	12.191	0.001
Enmeshed	16.040	5.103	13.650	4.159	9.924	0.002
Rigid	17.910	5.156	16.270	4.209	4.573	0.034
Chaotic	17.420	5.535	15.140	4.204	7.949	0.005
Communication	28.690	10.188	36.190	8.189	24.753	< 0.001
Satisfaction	26.910	10.334	34.240	8.676	22.453	< 0.001
*Attachment*						
Anxiety	0.400	2.864	-0.650	3.143	4.965	0.027
Avoidance	2.080	3.908	-0.020	3.722	11.885	0.001

Cluster 1 = High Distress / Low Relational Functioning; Cluster 2 = Low Distress / High Relational Functioning

## Discussion

This study offers two primary contributions to the literature on university counselling users. First, we identify a clear, medium-sized pattern of associations linking student distress to relational context. More than half of the sample scored above the CORE-OM clinical cut-off, and higher distress co-occurred with both insecure attachment (anxiety and avoidance) and unbalanced family functioning (disengagement, enmeshment, rigidity, chaos), alongside lower family cohesion, flexibility, satisfaction, and communication. Taken together, these concurrent associations support a systemic view in which the severity of current symptoms is meaningfully embedded in students’ patterns of relatedness—both in the family of origin and in attachment dispositions that shape help-seeking, affect regulation, identity development, and alliance formation [38, 39, 64]. Clinically, the observed correlation structure provides a coherent template for shared case formulation and for focusing the counselling intervention. When a student presents with elevated CORE-OM scores, the inverse associations with family cohesion, flexibility, satisfaction, and communication indicate immediately actionable foci of intervention that link distress with family relationships [65, 66]. In parallel, the positive associations between distress and both attachment anxiety and avoidance underscore the need to establish relational safety and predictability from the outset, with explicit attention to micro-processes of engagement and the rupture-and-repair of the therapeutic alliance [41–43, 67, 68]. In sum, the correlation pattern suggests that distress in this cohort reflects not only intrapsychic strain but also relational processes. This supports a brief, focused approach that targets interpersonal mechanisms aligned with our findings—principally communication effectiveness and boundary-setting—both of which are associated with family functioning and symptom reduction in student samples [65, 66].

The second main contribution is person-centred: a two-cluster solution differentiating students with High Distress/Low Relational Functioning from those with Low Distress/High Relational Functioning. The High-Distress cluster combined elevated CORE-OM scores with lower family cohesion and flexibility, lower satisfaction and communication, higher disengagement/enmeshment/rigidity/chaos, and more insecure attachment (both anxiety and avoidance). The Low-Distress cluster displayed the complementary pattern: sub-clinical distress, stronger balanced family functioning, fewer unbalanced patterns, and lower attachment insecurity. This profile difference accords with classic separation–individuation work in college populations, which consistently distinguishes “strained, dependent or engulfed” subgroups from “healthy separators” with better psychological adjustment [69]. The added value here is that the profiles integrate family-systems variables and attachment with a clinical outcome index, offering a compact decision aid for tailoring care. At the service level, the profiles map naturally onto a stepped-care architecture. Students resembling the Low-Distress/High-Functioning profile can receive low-intensity brief care (fewer sessions) centred on psychoeducation and skills rehearsal, with outcome monitoring. Students in the High-Distress/Low-Functioning profile merit more structured modules (e.g., 6–8 sessions) that combine reflection on family functioning and its impact on self-regulation and identity, targeted self-compassion work, and, where indicated, activation of referral pathways for comorbidity or acute risk [65].

Another important finding concerns the link between family communication and mental-health outcomes. Evidence from U.S. undergraduate samples indicates that disengaged family patterns undermine communication, which in turn relates to poorer perceived mental health; enmeshment follows a partially distinct pathway, although communication remains linked to mental-health outcomes [66]. This pattern is consistent with family-systems-informed accounts—such as Internal Family Systems—that view internalised relational roles as shaping self-regulatory “parts” of the mind [70, 71]. Within this framework, students from disengaged contexts may be more likely to internalise tendencies toward emotional self-neglect and reduced help-seeking, whereas students embedded in more balanced family climates may show greater communicative efficacy and access to supportive exchanges. Our correlations—distress co-occurring with lower cohesion/flexibility/satisfaction/communication and higher disengagement/enmeshment/rigidity/chaos — are therefore compatible with a model in which communication operates as a proximal relational mechanism linking family functioning to psychological distress and self-regulation strategies in university populations.

Clinically, for High-Distress students who also endorse low cohesion/flexibility, counselling can set realistic, skill-focused goals that combine strengthening self-representation (active listening to the self, naming needs) and self-compassion as a corrective to self-neglect, alongside brief communication-skills practice (listening, conflict management, role-playing) as a feasible, low-dose intervention for campus services [66]. Moreover, our findings align with research showing a pathway from balanced cohesion/flexibility to better family communication, then to higher self-compassion, which relates to lower anxiety and depression; these indirect effects are comparable across genders [65]. Translating this mechanism into practice, High-Distress students may benefit from an intervention focused on helping them mentalise the links between current self-regulation strategies and formative family relationships (i.e., noticing how relational scripts reappear in coping).

Furthermore, attachment insecurity appears central both to risk and to help-seeking. In U.K. students, anxious attachment predicts help-seeking among those with distress, whereas avoidant attachment may suppress help-seeking unless distress is very high [41]. Complementary Italian data show that counselling attenders (vs. non-attenders) report higher distress and more insecure attachment, with robust attachment–psychopathology links in both groups [68]. In light of our High-Distress/Attachment-Insecure profile, intake should explicitly name and normalise relational-safety needs, clarify the setting (predictability, boundaries), and anticipate avoidance-driven dropout by setting early, concrete, developmentally relevant goals (e.g., building safer relationships, consolidating identity) and using short feedback cycles. Finally, the findings underscore the role of attachment security and balanced family functioning in students’ psychological well-being. This suggests that fostering attachment security and emotion regulation should be integrated into psychological interventions for university students [72]. In addition to individual interventions such as cognitive-behavioural therapy and mindfulness-based programmes [73], group formats may be beneficial in enhancing acceptance, belongingness, and social support [22].

### Limitations and future directions

The findings of this study should be interpreted in light of several limitations.

First, the use of a self-selected sample from a single center limits the generalisability of the findings, as participants were students who had actively accessed psychological support and may therefore differ from the broader population of university students, including those who experience psychological distress but do not seek help. Moreover, because no information was collected on students who did not complete the assessment, differences between participants and non-participants cannot be ruled out. The marked predominance of female participants should also be considered when interpreting the results, as gender imbalance may have influenced the observed psychological and relational profiles. Future research should therefore replicate these findings in larger and more gender-balanced samples, including students from multiple universities and counselling services, as well as comparison groups of students who do not access psychological counselling. This would help clarify whether the profiles identified in the present study are specific to help-seeking students or reflect broader patterns within university populations.

Second, reliance on self-report measures should be highlighted. Although self-report instruments are widely used in psychological research and allow the efficient assessment of subjective distress, family functioning, and attachment-related dimensions, they may be affected by response biases, including social desirability, limited self-awareness, or individual differences in the interpretation of questionnaire items. As a consequence, the findings should be interpreted as reflecting students’ subjective perceptions of their psychological and relational functioning rather than objective or externally validated indicators. Future studies should integrate self-report questionnaires with clinician-rated assessments, observational procedures, or external-report measures, in order to obtain a more comprehensive and multi-informant assessment of students’ psychological and relational functioning. Such an approach would help determine whether the identified profiles are also observable across different sources of information.

Third, the associations among psychological distress, family functioning, and attachment should therefore be interpreted as concurrent relationships rather than as evidence of directional or causal pathways. It is not possible to determine whether less adaptive family functioning and insecure attachment patterns contribute to higher psychological distress, whether distress influences students’ perceptions of their relational context, or whether these dimensions interact over time. Future longitudinal studies are needed to examine the temporal stability of the identified profiles and to clarify whether family functioning and attachment-related variables predict subsequent psychological distress, counselling engagement, dropout risk, symptom trajectories, or response to intervention.

Fourth, although the cluster solution was theoretically coherent and aligned with established person-centred patterns, it remains exploratory and sample-specific until replicated in independent samples. The two profiles should therefore be interpreted as preliminary and context-sensitive rather than as definitive evidence of stable subgroups among university students. Future research should replicate the cluster solution in larger independent samples and test its robustness across different institutional, sociocultural, and clinical contexts. Measurement-invariance testing across relevant groups, such as gender, degree program, and clinical status, would also help determine whether the instruments and the resulting profiles operate similarly across different student populations.

### Practical implications

Despite the limitations, the study has relevant practical implications. The identification of distinct psychological and relational profiles among students accessing counselling services may help university counselling centres move beyond a symptom-only assessment model. Brief screening procedures that integrate psychological distress, family functioning, and attachment-related dimensions could support the early identification of students with more complex emotional and interpersonal difficulties. Students showing high distress and low relational functioning may benefit from more structured assessment, closer monitoring, and interventions focused not only on symptom reduction but also on interpersonal functioning, emotion regulation, and continuity of help-seeking. Conversely, students with lower distress and more adaptive relational functioning may benefit from brief preventive or supportive interventions. From this perspective, the findings may inform stepped-care models and contribute to tailoring psychological support within university counselling services.

## Conclusions

This study indicates that psychological distress among university counselling users is closely intertwined with family functioning and attachment, and that these relational dimensions cohere into two clinically meaningful profiles. Beyond describing associations, the person-centred solution offers a pragmatic framework for care planning: low-distress students can be supported with low-intensity psychoeducational approaches, whereas high-distress students benefit from structured brief modules targeting communication, boundary-setting, emotion regulation, and self-compassion, with referral pathways activated when complexity or acute risk is present. Taken together, the findings argue for mechanism-focused, stepped interventions that translate relational assessment into specific, teachable skills.

## Data Availability

Data presented in this study are available on request from the corresponding authors.
